# Hygienics of Temperance, or Water and Alcohol Contrasted on the People Proper

**Published:** 1854-10

**Authors:** Samuel A. Cartwright

**Affiliations:** New Orleans, Late of Natchez, Canal Streeet, New Orleans


### HYGIENICS OF TEMPERANCE, OR WATER AND ALCHOHOL CONTRASTED ON THE PEOPLE PROPER.

BY SAMUEL A. CARTWRIGHT, M.D., NEW ORLEANS, LATE OF NATCHEZ.

[From the Boston Medical and Surgical Journal.]

When the writer had the honor to receive the Rev. Dr. C. K. Marshall's communication, calling on him for the physical effects of alcoholic beverages on the different classes of people, who had come under medical observation, he was about visiting Natchez, his old place of residence. He looked over his account books of the year 1823 to 1828, and made a list of all those persons on whom he had attended at that period, as physician, and whose habits he knew. He took the list with him to Natchez, and added to it such other persons among his former acquaintances whom he recollected sufficiently well to classify on the question of temperance or intemperance. After making the classification, he submitted it to several of the older inhabitants for emendation and correction. The list contained 790 names—male adults of Natchez and its vicinity. As the women are all temperate, no notice was taken of them in collecting the statistics; nor of the minors, but only of the people proper, the true sovereigns.

Classified—the list stood: temperate, 330; intemperate, 460. All those who were in the habit of using alcoholic beverages, between meals, whether they ever got intoxicated or not, were classed among the intemperate. Whereas about 80 of those, classed among the temperate, occasionally indulged in a frolic or in what is called a social glass, in holiday times or at some particular celebration or convivial entertainment—thus reducing the number of the strictly temperate to about

250. Great assistance in making the classification was derived from the recollection of those who patronized grog-shops, bar-rooms and exchanges—being of that class which could scarcely be ten minutes together without some one of the company proposing to "treat," as it was called, or to "take a drink." With this class, a political, moral or religious discourse of an hour or two in length, was generally considered a bore, as being too long between drinks. All these are classed among the intemperate, although, perhaps, not as many as an hundred of the 460 were in the habit of getting intoxicated, and the remainder seldom or never. Yet they daily partook of ardent spirits, as regularly as their meals, and much oftener. They contributed very little to the support of the medical faculty, and were remarkably careless and regardless of their health. For minor ailments, such as colds, colics, diarrhœa, &c., instead of calling in a physician immediately as the temperate class did, they generally took an extra glass or two of their usual stimulating potations, often adding spices, and taking it hot instead of cold—thus approaching very nearly to the Thomsonian system of medication. If they could force a sweat, relief was apt to follow—or the over-charged stomach would reject its contents, the stimulating potations acting as an emetic and carrying off the bile. Hence, from their seldom calling in medical aid, the impression was made upon the public mind, that the use of spirituous liquors prevented disease. This delusion was confirmed by the fact that the temperate were so often sending for physicians. Their senses not being stupified, they felt any derangement in their systems, more evidently than those who were under the benumbing effects of alcohol, which in large quantities palsies the sentient system, like ether or chloroform. It was only, therefore, in cases in which alcoholic drinks failed to give relief, that medical advice was sought. It was often sought too late, and this class of people were very apt to die from maladies the temperate would very readily recover from, both from thus applying earlier, being more tractable and manageable, and having their blood in a better state. If a physician succeeded in curing the intemperate of the most dangerous diseases, he would often get neither pay nor thanks—as they did not know how ill they were. They came out of a serious illness, as they did out of a drunken frolic, with a forgetfulness of the past. If he were unable to succeed in curing them, his reputation suffered, as the friends of deceased were apt to blame him for negligence or want of skill, pointing to this person or that who recovered from a much worse condition. As a general rule, they never recover from yellow fever, as about nine out of ten of the temperate do. Pneumonia, erysipelas, and fevers connected with chronic inflammation of the bowels or liver, carry most of the intemperate off.

How many of the 460 were living, after the lapse of a quarter of a century, was a question that it was important to ascertain. On making inquiry of the old inhabitants of Natchez, not more than a couple of dozen could be found or heard from. The greater part were known to be dead—and the balance had gone, no one knew where. Nor were there any sugar hogsheads or cotton bales rolling into the southern emporium to indicate the whereabouts of the missing. Champomier, in his "Statement of the Sugar Crop," has given a list of 1481 plantations, making 368 millions of pounds of sugar and 25 7-10 millions of gallons of molasses in 1852, with the names of the owners. None of the missing were found on that list. Nor did the cotton commission merchants know anything of them. Whereas, nearly all of the temperate class who have removed from Natchez and its vicinity, are on the list of either the cotton or sugar brokers. For the remainder of his old friends and patrons of the temperate class, the writer, on his arrival in Natchez, found it almost unnecessary to inquire how many of them were still living, after a quarter of a century had passed by. For time seemed to have rolled backward, and there they were before him to speak for themselves. They were all living, excepting a few who had died from old age and accidents. According to the

Carlisle tables, 330 individuals, at the average age of 25 years, would lose, in a quarter of a century, 83 of their number. Whereas only 68 have died in from 25 to 30 years; and their average age was more than 25 years. Yet in the average time of 27 ½ years, a smaller number had died, than the natural decrease in the most healthy countries. Speaking in general terms, it really did appear, that they were not only all living, but had all the money. Certainly, in proportion to their numbers, the lion's share of the wealth of the country belongs to them. If their property were equally divided, each would have upwards of one hundred and fifty thousand dollars for his share. The statistical figures made it much more. Water, as a beverage, is not only a good life-preserver, but it pays well. When first known to the writer, 25 to 30 years ago, many of them were serving out their probation under Temperance, as Jacob served Laban. They were rewarded with the most accomplished Leahs and the fairest Rachels of the land—women who had too much good sense, polish, education and discretion to trust their persons and property to the whims, caprices, inconsistencies snd uncertainties incident to the character of all those, no matter how plausible and attractive in other respects, who had not been trained, by the self-denying disciple of Temperance, to habits of sobriety, industry, constancy, consistency and stability. Poor they were, but having served out their probation and stood up to their principles through good and through evil report, they had that which soon made them more than rich—a character for sobriety, honesty, industry and attention to business, worth more than wealth. Some few jumped immediately into the possession of large estates by matrimonial alliances, while the greater number slowly rose to the attainment of princely possessions by industry and economy of money and time, coupled with a generous liberality of expenditure in praiseworthy objects—having always at hand, fives, tens, hundreds, and in some instances thousands and tens of thousands to build schools, colleges, churches, and for internal improvements; but never a dime to spend to support those drinking establishments, where time is wasted, morals corrupted, health impaired, broils engendered, and the bowie knife, the judge, jury and executioner. The four hundred and sixty patronizers of such places of resort, 25 to 30 years ago, have nearly all passed away, leaving only here and there an infirm, sickly descendant to tell that any of them had ever been. Even the *Pere la Chaise* of Natchez contained scarcely a stone, that the writer could find, erected to the memory of any of the 460. The only place where the existence or non-existence of any of them, with a few exceptions, could be found, was the *sexton's book.* That did not tell of all who had died, but only those who had died at home. But it told of more than the larger half—enough and more than enough to draw a strong contrast between water and alcohol as beverages when used by the people at large—and to prove that intemperate habits, in this southern climate, cause a frightful waste of human life. The marble told of nearly all of those few among the 330 temperance men, who were not living; while the streets of the city declared that the marble was mistaken—that they were not dead, as it said, but were living life over again, in the shape of a new generation or two of an active, healthy and wealthy posterity; having to tread cautiously, as their fathers did, to avoid the pits, with concealed mouths, at the corners of the streets and near by all public places of resort, into which many of them are, ever and anon, dropping.

The swarms of children, however, which the writer saw in every direction, pouring out of the school houses built by his old temperate acquaintances and patrons, particularly that large one, called the Institute, donated to the public by that best of men, Alvarez Fisk, gave evidence of a latent principle in physiology which would form a fine study for theologians and statesmen—viz., that the temperate in all things are sufficiently prolific soon to spread a progeny of the hale and the healthy, the civilized and the christianized, over the earth's broad surface, provided that progeny be prevented from falling into intemperate habits, which can only be done by protecting them from the ethyl breath—mesmerizing the will—exhaling from the numerous grog-shops spread over this fair land.

The converse of the same physiological principle is, that a nation of intemperate people will soon become extinct, if both sexes be so; short-lived, rheumatic and consumptive, if only one be. The Indian nations, one after the other, are disappearing—both sexes being intemperate. The writer practiced medicine two years near the Indian line, and observed that drunken squaws were seldom or never troubled with papouses—nearly all the children belonging to the temperate Indians. The black race, like the red, diminish faster than they multiply in the free States, Hayti, Canada, Sierra Leone, and wherever they have free access to spirituous liquors. They hold their own in the wilds of Africa, where they can get none. They multiply rapidly in those countries where their will, like children, is under restraint—the government paternal, and sufficiently kind to protect them from that great enemy of mankind, alcohol. Their own will is too weak, with the scent of that substance in their wide nostrils, to prevent them from leaving all industrious pursuits, and the places of religious and moral instruction, for the haunts of dissipation, from which moral suasion cannot draw them. The exceptions prove the general rule. In the depopulated neighborhoods of Eastern Virginia, the ruins of a distillery were often found in the boyhood rambles of the writer. In those days the Ethiopian race were accused of being the curse of Virginia and America, and great numbers of whites ran away from their negroes to the far West. But at that early day a strong impression was made upon the writer's mind, that the thing which made good men bad, and free negroes worse than slaves, was surely the cause of the misfortunes of Virginia, as nearly all the evil he had even seen, sprang from that source alone. He resolved never to drink ardent spirits, and when he grew to be a man to do what little he could to abate the evil. The Rev. C. K. Marshall's communication, calling for his observations of the effects of alcoholic beverages on the different classes of people—white, black and red—reminded him of his former determination, and he only regrets his inability to do justice to the subject. The facts that he has adduced prove, that the advocates of alcohol have no right to seek shelter or protection in the science of medicine.

The means that physiology would suggest to protect the offspring of the prolific temperate from the evils of the traffic in ardent spirits will, fortunately, not only give the largest liberty to the greatest number, but will go a step beyond—a step of true republican progress—and give the largest liberty to the whole number, consistent with the health, peace and happiness of the various classes composing the whole number. This liberty they cannot have, while pits are everywhere dug and left open for their children to fall into, and while public places, private walks, hotels and thoroughfares are infested with lawless, dangerous madmen.

But there are other matters, besides mere temperance, which have to be taken into consideration to account for so much health, wealth, longevity and prosperity in a climate as hot as that of Natchez. The field and out-door work is performed by a class who delight to expose their persons to a bath of bright solar light, hot enough to blister the skins and inflame the blood of the other class. In the shade the temperature is nearly the same, in the summer months, at the North and the South. But the thermometer, in the shade, gives no idea of the insufferable heat of a cane or cotton field in the summer months at noon.

Another important matter—the water, in that section, is not only good, but it is probably the best in the United States; being mostly rain water, collected at the proper time and manner, in well-constructed cisterns, and kept cool and freed from organic and fermentable matter. To draw a fair contrast between water and alcohol, the water should be good. A very large proportion of the spring, well and river water, throughout the Union, is very impure. The summer rains contain much more organic and fungoid matter in the North than at Natchez or New Orleans. But at the latter places a few fish in the cisterns are very useful in purifying the water. Those who are now employed in preparing the various kinds of alcoholic beverages, could be better employed in filtering and freeing water, to be used for drinking and culinary purposes, of all impurities, as that of lead and other mineral impregnations, dead animalcules, fermentable substances, earthy and alkaline salts. All such impurities injure the teeth and deteriorate the blood—a fluid so nicely balanced between chemical and vital forces, that any deteriorating cause diminishes its vitality, as alcohol does, and lays the foundation for diseases of various kinds. The purification of water by filtration, surface action and other means, would not only be a good business for all those now engaged in dispensing and preparing alcoholic drinks, but would atone for much of the evil they have already done.

Canal Street, *New Orleans*, *July 2d*, 1853.

